# Household Labour During the Workday in Remote Work Contexts. Who Integrates It, and Why?

**DOI:** 10.5334/pb.1477

**Published:** 2026-07-01

**Authors:** Nathan Pudles, Marine Willeput, Sabine Pohl, Catherine Hellemans

**Affiliations:** 1 Department of Work and Consumer Psychology, Université libre de Bruxelles, Belgium

**Keywords:** Remote work, border theory, social roles theory, household labour, fractional polynomials

## Abstract

Remote work is often presented as a means to balance out work and family domains, by giving the ability to integrate the two domains. Using border theory and social role theory as a framework, the present study aims to uncover the relationships of autonomy, job demands, work engagement, and the involvement with domestic tasks, with doing household labour during the workday (HLW), and how these relationships may differ depending on gender. The study was conducted among a sample of 1309 workers (65.77% women), who worked remotely at least once a week. Hypotheses were tested with a logistic regression and fractional polynomials. Results were in contradiction with the hypothesized relationships. Job demands and work engagement increased the probability of doing HLW. Autonomy decreased the probability of doing HLW. Involvement in domestic tasks was non-linearly associated with HLW. Specifically, workers who had to deal with domestic tasks mostly by themselves were more likely to resort to HLW, whereas others were less likely to do so. The relationships of autonomy, job demands, work engagement and IDT with HLW did not differ depending on gender.

## Introduction

With the fast development of information and communication technologies (ICT) and since the Covid-19 pandemic, remote work has become a staple in many organizations, notably with the implementation of hybrid work, a practice that involves switching between working remotely and working from the office (Lauring & Jonasson, 2025). In the European Union, the share of workers working from home at least sometimes rose from 13.6% to 23% between 2018 and 2025 (Eurostat, 2025). In Brussels, remote work practice was already more established prior to the Covid-19 pandemic: it rose from 17% to 36% between 2018 and 2025 (Duchêne et al., 2024; SPF Mobilité et Transports, 2025). More workers can now benefit from greater autonomy and more flexible boundaries between work and family domains, which could help them managing household labour (e.g. childcare, cleaning, grocery shopping…) by integrating it into the workday (Brumley & St. George, 2025; Choi, 2020; Van Der Lippe & Lippényi, 2020). As such, remote work is often presented as a means to balance out work and family domains (Blom et al., 2025).

However, there are some caveats. The extent to which remote work can help workers combining job demands and household labour to the best of their needs can depend on the organizational context (Van Der Lippe & Lippényi, 2020). For instance, remote workers can end up working outside of regular workhours, notably when exposed to high levels of job demands, leaving them with little room to adapt their work domain to household labour (Aloisi & De Stefano, 2022; González-Fernández et al., 2025; Van Der Lippe & Lippényi, 2020).

Moreover, although remote work can help managing household labour, it can also contribute to gender inequalities, both in the family and the work domain. Indeed, household labour is deeply gendered, and social roles set women in heterosexual relationships as the primary homemakers, thus having to deal with most household labour (Eagly et al., 2000). Remote work can help women to dedicate more time to their paid work, all the while maintaining their output in household labour, but it can also increase the amount of household labour they are exposed to, from their own initiative or due to their partner’s expectation (Clar-Novak, 2025; S. Wang & Cheng, 2024). This was particularly salient during the Covid-19 pandemic: mothers working remotely in heterosexual relationships had to take care of the bulk of household labour, which significantly increased, notably due to children having to stay at home, whereas their partner often chose to increase their investment in the work domain (Chatot et al., 2023; Clar-Novak, 2025).

Although there has been substantial research addressing the conflicts between the work and family domains that can arise when working remotely (e.g. Beckel et al., 2023; Ferri et al., 2018; Gohoungodji et al., 2023; Smith et al., 2021; B. Wang et al., 2021), the factors associated with the integration of the family domain to the work domain are yet to be understood outside of the Covid-19 pandemic context (Chatot et al., 2023; Clar-Novak, 2025; Desjardins et al., 2024). Indeed, during the Covid-19 pandemic, remote work had been enforced and schools had shutdown amidst the successive lockdowns, thereby substantially distorting the characteristics of the work and family domains (Desjardins et al., 2024). Now that remote work has become part of the new normal in many organizations in the post Covid-19 context (Eurofound and European Commission Joint Research Centre, 2024), understanding what can drive the integration of the family and work domains would bring an important contribution to the literature on remote work.

Therefore, using border (Clark, 2000) and social role theories (Eagly et al., 2000), the first objective of this study is to outline the relationships of work characteristics and personal attitudes with the performance of household labour during the workday (HLW) when working remotely. The second objective is to assess how gender can influence the relationships of work characteristics and personal attitudes with HLW.

## Household Labour During the Workday, Work Characteristics and Personal Attitudes

According to the border theory (Clark, 2000), work and family domains are separated by physical, temporal and psychological borders. The physical border refers to where domain-specific tasks are performed, the temporal border refers to when domain-specific tasks are performed, and the psychological border refers to the rules defined by the individual about when and where domain-specific behaviours and thinking patterns are appropriate or not.

These three borders can be subject to permeability and flexibility. The level of flexibility and permeability define the strength of the borders. A key distinction to be made between flexibility and permeability is that flexibility implies to have a certain degree of agency over how element of one domain can flow to the other domain, in accordance with the rules set by the individuals. Permeability pertains to the intrusion of one domain in the other domain, thereby bypassing the rules established by the individuals (Schieman & Glavin, 2008). Whereas strong borders are impermeable and inflexible, weak borders can let individuals attune the two domains when needed (flexibility) but can also let elements of a domain spillover to the other domain (permeability). Weak borders can increase the risk of generating work-to-family and family-to-work conflicts (Clark, 2000; Olson-Buchanan & Boswell, 2006). These two types of conflicts are associated with mental ill-health, life dissatisfaction and turnover intentions (Dettmers, 2017; Mesmer-Magnus & Viswesvaran, 2005; Zhang et al., 2012).

Border strength depends on domain-specific characteristics, as well as personal attitudes towards both domains (Allen et al., 2014; Clark, 2000; Kossek et al., 2012). Moreover, border strength is asymmetrical; borders can be impermeable from one domain to the other, but the opposite may not be true (Allen et al., 2014). Kossek et al. (2012) found different border management styles, e.g. some individuals adapt their family domain to their work domain, allowing spillovers from the work domain to the family domain, but do not adapt their work domain to their family domain. These individuals would bring work back home, or respond to work-related messages after the end of the workday or during the weekend, but would not adapt their work schedule to family demands (Hochschild, 1997). They integrate the work domain to the family domain, but do not integrate the family domain to the work domain; the borders of their family domain are flexible and permeable to the work domain, but the borders of their work domain remain strong (Allen et al., 2014). Others can adapt their work domain to their family domain, but do not adapt their family domain to their work domain (Kossek et al., 2012). They would respond to family demands during the workday, such as doing household labour during the workday (HLW), but would not respond to work-related demands after the end of the workday (Chung & Booker, 2023; Genadek & Hill, 2017). They integrate the family domain to the work domain, but do not integrate the work domain to the family domain; they have strong family domain borders but flexible and permeable work domain borders (Allen et al., 2014).

When working from home, the physical border is thin or inexistant, as individuals work in a home office room or do not have a dedicated room at all (Gálvez et al., 2019). Though it can give more flexibility to perform HLW, it also facilitates the spillover of one domain to the other (Clar-Novak, 2025; Smith et al., 2021). It becomes easier for workers to extend their worktime outside of traditional workhours, which can generate interruptions from the work domain to the family domain (González-Fernández et al., 2025; Wöhrmann & Ebner, 2021). Interruptions by the family domain can also arise during the workday (Chatot et al., 2023; Gálvez et al., 2019).

### Household Labour During the Workday and Work Characteristics

Integrating the family domain to the work domain, namely by doing HLW, would depend on the strength of the borders of the work domain. HLW could occur by choice (flexibility) or due to the intrusion of elements of the family domain (permeability). Although remote work has the potential to increase the flexibility and the permeability of the borders of the work domain, *how* this potential is enacted by workers would depend on work characteristics and personal attitudes that could contribute to determine the flexibility and permeability of the borders of the work domain.

Establishing flexible work domain borders when teleworking, to let elements of the family domain enter the work domain, by doing HLW, could be facilitated by having greater work autonomy (Metselaar et al., 2023). Work autonomy can be defined as the amount of decisional latitude one has over the schedule of their work, the decisions they make, and how they perform their tasks (Morgeson & Humphrey, 2006). Whilst remote work tends to be associated with greater work autonomy (Schulze et al., 2024), workers can be digitally monitored when working remotely, or feel pressured to be permanently available via ICT means (Afota et al., 2023; Aloisi & De Stefano, 2022). Having little to no autonomy when working remotely would complicate the adaptation of the work domain to the family domain, whereas this adaptation is precisely seen as a benefit associated with remote work (Vayre et al., 2022). By enabling workers to better determine the organization of their workday, autonomy could allow them to do HLW.

H1. Autonomy is positively related to HLW.

By contrast, being exposed to high levels of job demands, such as high workload, pressuring deadlines or task interruptions, can constrain workers to focus their resources on their work (Paškvan & Kubicek, 2017). This could give workers little leeway to adapt the borders of the work domain to integrate the family domain (Tammelin et al., 2017), as heightened job demands can be a means, for organizations, to ensure that their workers are busy with work-related activities during the workday, rather than “slacking off” (Aloisi & De Stefano, 2022; Telford & Briggs, 2022). Moreover, switching between work tasks and household labour requires an investment in self-regulatory resources, which are already solicited when being confronted to high job demands (Johnson et al., 2017; Leroy et al., 2020). As self-regulatory resources are limited, their investment in attention focus and effort to manage job demands, and in task switching to do HLW, could expose workers to ego depletion (Baumeister et al., 2024; Johnson et al., 2017). Therefore, implementing flexible work domain borders could be too costly when exposed to high job demands, and doing HLW could put workers who have high job demands at risk of experiencing family-to-work conflicts and a decrease in their work performance (Clinton et al., 2020; Nohe et al., 2014). Thus, job demands could be considered as an obstacle to doing HLW.

H2. Job demands are negatively related to HLW.

### Household Labour During the Workday and Personal Attitudes

The attitude of workers towards their work can also influence the enactment of HLW (Kossek et al., 2012). Workers who are engaged in their work are devoted to, absorbed in and envigored by their work (Bakker & Demerouti, 2008). Engaged workers could either be strongly committed to their work role and invest most of their personal resources in their work, at the expense of their family role (Chen & Huang, 2016; Halbesleben et al., 2009), or dedicated to maintain strong borders between the work and family domains, with enough personal resources to allocate to both domains (Gálvez et al., 2019). Either way, we could expect that remote workers who are deeply engaged in their work would have strong work domain borders and tend to not integrate their family domain to their work domain, notably by avoiding doing HLW.

H3. Work engagement while working remotely is negatively related to HLW.

Moreover, remote work is often presented as a means to improve the balance between work and family domains, as it can enable workers to integrate aspects of the two domains and thus cope more easily with both job demands and household labour (Blom et al., 2025). However, whilst high job demands can spillover to the family domain, household labour can also spillover to the work domain (Chatot et al., 2023; Michel et al., 2011). A worker who has a high level of involvement in domestic tasks (IDT) can experience intrusions of household labour during their worktime, as they would have to take care of most of the domestic tasks (Desjardins et al., 2024). Furthermore, similarly to job demands, doing household labour requires an investment of resources, such as time and energy (S. Wang & Cheng, 2024). Investing resources to manage job demands can reduce the resources necessary to deal with household labour, especially with a high IDT, which could result in work-to-family conflicts (Voydanoff, 2005; Yucel & Chung, 2023). Thus, for workers with a high IDT, HLW could also be a way to prevent further resource loss and to respond adequately to demands arising from the family domain, when they are working remotely.

H4. Involvement in domestic tasks is positively related to HLW.

## Household Labour During the Workday and Gender Roles

Social role theory posits that gender is a social construct that determines distinct roles, each having typical characteristics and behavioural expectations; these expectations can vary across societies. In Western capitalist societies, the male gender role is to be agentic and a breadwinner, whilst the female gender role is to be communal and a homemaker/care provider (Eagly et al., 2000). Even though women are now integrated into the labour force in Western societies, gender role expectations remain the same: women are expected to take care of the family domain, and men are expected to take care of the work domain (Treas & Tai, 2016). As such, household labour is mainly taken under the responsibility of women, with working women being confronted to a double shift, between their paid and unpaid (domestic) work (Brumley & St. George, 2025; Hochschild & Machung, 1989). This was particularly salient during the successive lockdowns due to the Covid-19 pandemic (e.g. Chatot et al., 2023; Clar-Novak, 2025; Collins et al., 2021; Hanzl & Rehm, 2023; Parry, 2025).

A key perspective to consider when taking both border and social role theories into account is domain centricity. Kossek et al. (2012) found that border management can depend on whether individuals are work-, family-, or dual-centric. Regarding gender roles, and their underlying behaviours and expectations (Eagly et al., 2000), it could be expected that women would tend to be more family-centric, and have more permeable and flexible work domain borders, whilst men would be more work-centric, and have more permeable and flexible family domain borders. Indeed, men tend to report more family-to-work conflict than women when the family domain spills over to the work domain (Eddleston & Mulki, 2017). In the contrary, women who work remotely tend to report more work-to-family conflicts than men when work spills over to the family domain (Desjardins et al., 2024; Nsair & Piszczek, 2021). Therefore, we could expect that HLW is done by more women than men.

H5. Women are more likely to do HLW than men.

Moreover, there is a difference in border management for the work domain between fathers and mothers occupying professional and managerial positions. Though these positions tend to have a high level of autonomy, they are also typically associated with high job demands (Schieman & Glavin, 2016). Nonetheless, Chung and Van Der Horst (2020) found that mothers occupying these positions did not allow work to interrupt the family domain. Genadek and Hill (2017) found that working mothers that used flexible working arrangements, such as schedule control or remote work, would spend more time with their child; for fathers, time and place autonomy had no relationship with the time they spent with their child. Similarly, S. Wang and Cheng (2024) found that flexible working arrangements were related to an increase in household labour for women but not for men. However, it also occurs that partners do not respect the borders set by remote workers and expect a higher involvement in domestic tasks from the latter. These patterns are most common when the remote worker is a woman, in a heterosexual relationship (Eddleston & Mulki, 2017; Gálvez et al., 2019; Parry, 2025; S. Wang & Cheng, 2024). Furthermore, social roles persist even when women are confronted to high job demands or are engaged in their work; they are still expected to take care of most household labour, which could require them to adapt the flexibility of the borders of the work domain to do HLW. On the contrary, men who are confronted to high job demands or are engaged in their work tend to prioritize their work, which can come at the expense of household labour (Clar-Novak, 2025; Eagly et al., 2000; Parry, 2025). Men also tend to overestimate their IDT (Geist, 2010), which could result in a weaker association between IDT and HLW for men than for women.

Thus, we expect that the previously outlined work characteristics and personal attitudes may have different relationships with HLW, depending on gender.

H6. Gender will moderate the relationships between autonomy, job demands, IDT and HLW, such as (a) the positive relationship between autonomy and HLW will be stronger for women than for men, (b) the negative relationship between job demands and HLW will be weaker for women than for men, (c) the negative relationship between work engagement and HLW will be weaker for women than for men, (d) the positive relationship between IDT and HLW will be stronger for women than for men.

**Figure 1:**
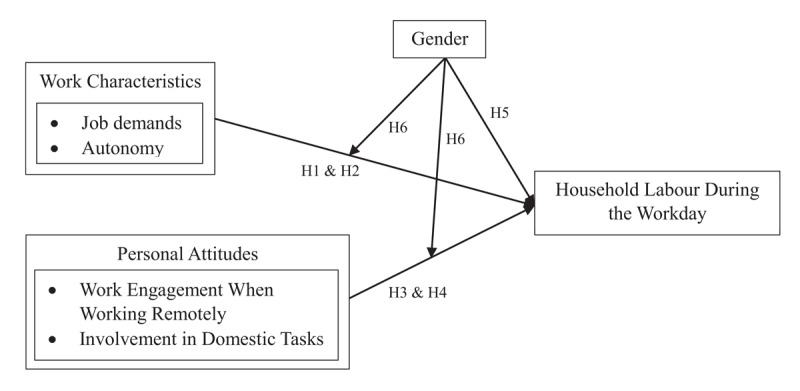
Hypothesized Relationships.

See Figure 1 for a graphical representation of the hypothesized relationships.

## Method

### Procedure and Participants

A cross-sectional survey was conducted in 2024 in Belgium among 1309 workers who worked remotely at least once a week. The survey was distributed via large organizations, who shared the survey among their employees, and through social networks, to reach workers from a greater diversity of organizations. The questionnaire was made available in French, Dutch and English, the first two languages being the official languages in Belgium. We used the translation/back-translation procedure (Brislin, 1970) for the scale that were not validated in French or Dutch. Participation was anonymous and on a voluntary basis.

65.78% of respondents were women, 81.1% did not do HLW, most of the respondents had a master’s degree (56.16%), 53.45% did not live in the Brussels Capital Region and 76.85% worked in large organizations (>250 workers) (see Table 1). Most respondents answered to the questionnaire in French (94.04%); the rest answered in Dutch (5.96%).

**Table 1:** Sample Description.


	%	*n*

HLW (ref = no)	18.9	248

Gender		

Women	65.78	861

Men	34.22	448

Education degree		

Lower secondary	0.90	11

Upper secondary	8.49	104

Bachelor’s	22.94	281

Master’s	56.16	688

Doctoral	9.80	120

NA	1.71	21

Living area		

Brussels Capital Region	46.55	608

Outside of Brussels	53.45	698

Organization size		

Small (<50 workers)	10.54	138

Medium (50 to 250 workers)	12.61	165

Large (>250 workers)	76.85	1006


Note. N = 1309.

Workers were informed of the research objectives, voluntary participation and confidentiality guarantee prior to their completion of the questionnaire. The research was examined and approved by the ethical committee of the university of the researchers.

### Measures

*Job demands* were measured with five items from the effort dimension of the Effort-Reward Imbalance questionnaire, excluding the item assessing physical demands (Siegrist et al., 2004). The items were measured on a Likert scale ranging from 1-*strongly disagree* to 4-*strongly agree*. Item examples are “I have constant time pressure due to a heavy workload” and “I have many interruptions and disturbances while performing my job”.

*Autonomy* was measured with three items from the decisional latitude dimension of the Job Content Questionnaire (Karasek et al., 1998). Items were measured on a Likert scale ranging from 1-*strongly disagree* to 4-*strongly agree*. Items were “My job allows me to make a lot of decisions on my own”, “In my job, I have very little freedom to decide how I do my work” and “I have the opportunity to influence how my work is carried out”.

*Work engagement while working remotely* was measured with the Utrecht Work Engagement Scale-3, in three items (UWES-3; Schaufeli et al., 2017). Respondents were asked to respond to the items based on how they estimated their work engagement when they work remotely. Items were measured on a Likert scale ranging from 1-*strongly disagree* to 7-*strongly agree*. Items were “I am immersed in my work”, “I feel bursting with energy” and “I am enthusiastic about my job”.

*Involvement in domestic tasks* was assessed with the item: “How do you see your contribution to household chores (housework, shopping, DIY, childcare…)?”, ranging from 1-*I hardly do anything* to 10-*I do almost everything*.

*Household labour during the workday* was measured with the item: “Do you take advantage of the flexibility offered by remote work to carry out private tasks (housework, shopping, DIY, childcare…) during your working day?”. The responses were yes or no.

### Analyses

Analyses were conducted in R. Descriptive and scale reliability statistics were calculated with base R and the package *psych* (Revelle, 2007). McDonald’s omega (ω) was used to estimate scale reliability.

As the questionnaire was answered in French and Dutch, we tested the configural invariance, the invariance of the factor loadings (metric invariance), of the item intercepts (scalar invariance), and of the residuals (strict invariance) between the two languages using *lavaan* (Rosseel, 2012), to test whether the data could be pooled for the rest of the analyses. The difference tests between the less constrained and the more constrained models should be ΔCFI < .01 and ΔRMSEA < .015 to attest of the invariance of the model at the more constrained level (Maassen et al., 2025). We used robust maximum likelihood estimation for these analyses, as the indicators of job demands and autonomy are ordinal with four response categories (Li, 2016). Thus, we only report scaled chi-square values, and robust CFI, TLI, SRMR and RMSEA.

The factor scores of the latent variables were then extracted using the regression method, which should yield unbiased coefficient estimates as the dependent variable is observed (Devlieger et al., 2016). The reliability of the observed factor scores, relative to their latent variable counterparts, are reported (Liu et al., 2025).

Hypotheses were tested with a multiple logistic regression, using the generalized linear model command in R. We controlled for age, the number of cohabitants and remote work frequency, as these variables can affect the level of job demands or IDT, and how borders are enacted when working remotely (Fonner & Stache, 2012; Piszczek & Pimputkar, 2021; S. Zhang et al., 2020).

Following the recommendations of Hosmer et al. (2013), we tested for the presence of non-linearity in the relationships between the independent and dependent variables by using the multivariable fractional polynomials procedure, with the package *mfp2* (Kipruto et al., 2023). The procedure consists of fitting a two-terms fractional polynomial transformation of a covariate of powers (p_1_, p_2_), against a one-term fractional polynomial transformation of the covariate of power (p_1_), and the linear covariate (p = 1). A one-term fractional polynomial transformation of the variable x would be x^p1^ (FP1) and a two-terms transformation would be x^p1^ + x^p2^ (FP2). The powers p_1_ and p_2_ are determined among the given set of powers {–2, –1, –0.5, 0, 0.5, 1, 2, 3}, which covers a wide array of non-linear functions (Royston & Sauerbrei, 2008). The fractional polynomial transformation of the covariate should be included if it significantly improves the deviance of the model (Hosmer et al., 2013). The process is repeated for each continuous independent variable of the model.

We then tested the significance of the hypothesized moderations. Testing the significance of the interaction of fractional polynomial terms with a categorical term differs from testing the interaction of a linear term with a categorical term (Hosmer et al., 2013). In the latter case, the model with the interaction is comprised of the two terms and their interaction. The interaction is deemed significant when its coefficient’s *p*-value is below .05. In the former case, the model with the interaction is constituted of the categorical term and polynomial terms specific to each level of the categorical term. The significance of the interaction is estimated with a likelihood ratio test (Royston & Sauerbrei, 2008). Four models were compared: Model 1 has no interaction, Model 2 accounts for the moderation of job demands by gender, Model 3 accounts for the moderation of autonomy by gender, Model 4 accounts for the moderation of work engagement while working remotely by gender, and Model 5 for the moderation of IDT by gender. The standard errors of the four models were estimated from 5000 bootstrap samples of the data.

The fit of the final model was assessed with the Hosmer-Lemeshow test (*Ĉ*), adapted to our sample size (Paul et al., 2013), and the Stukel test, based on a likelihood ratio test (LRT; Hosmer et al., 2013). Both tests follow a χ^2^ distribution. A model adequately fits the data when the tests yield a non-significant *p*-value at the 5% level. The accuracy of the model was assessed with the area under the receiver operating characteristic (AROC) curve. The threshold for acceptable accuracy is AROC ≥ .7 (Hosmer et al., 2013).

Results were analysed with the average marginal effects (AME) of each variable in the model, with the package *marginaleffects* (Arel-Bundock et al., 2024), as the raw coefficients of logistic regressions can be difficult to interpret (Howell-Moroney, 2024). For a FP1 or FP2 transformation of a variable, the AME is conditional on the values of the variable, resulting in different AMEs for each level of the variable (Royston & Sauerbrei, 2008). Bias-corrected and accelerated confidence interval (95%CI) of the AMEs were estimated with 1000 bootstrap resamples of the model.

## Results

### Descriptive Statistics

Means and standard deviations of the continuous variables, and the correlation matrix of the variables included in the analyses are displayed in Table 2. The composite scores displayed adequate reliability. HLW correlated with most independent variables, except for gender and remote work frequency, which could imply that gender and remote work frequency will not have significant effects in subsequent analyses. Age and the number of cohabitants correlated with several of our variables, including HLW, attesting the relevance of controlling for them (Bernerth & Aguinis, 2016).

**Table 2:** Means, Standard Deviations and Correlation Matrix.


	*M*	*SD*	*MIN*	*MAX*	*CORRELATION MATRIX*						

					1.	2.	3.	4.	5.	6.	7.

1. Job demands	2.67	0.71	1	4	(0.85)						

2. Autonomy	3.37	0.61	1	4	0.16**	(0.71)					

3. Work engagement	4.93	1.20	1	7	0.21**	0.24**	(0.86)				

4. Remote work frequency	2.53	1.06	1	5	–0.05	0.03	0.07*	—			

5. IDT	7.42	2.01	1	10	–0.00	–0.00	0.03	–0.06*	—		

6. Gender	—	—	—	—	0.04	–0.06*^a^	0.10**^a^	–0.06^a^	0.25**^a^	—	

7. HLW	—	—	—	—	0.17**^a^	–0.16**^a^	0.12**^a^	–0.06^a^	–0.15**^a^	0.05^b^	—

**Control Variables**											

Age	42.12	10.79	18	70	0.15**	.08**	0.17**	0.04	0.12**	–0.04^a^	0.29**^a^

Cohabitants	2.89	1.32	1	10	0.04	0.06*	0.05	–0.01	–0.20**	–0.04^a^	–0.07*^a^


*Note*. McDonald’s ω of the composite scores are presented in the diagonal of the correlation matrix. Gender was coded as 0 = *men*, 1 = *women*; Flexibility use was coded as 0 = no, 1 = yes. IDT = Involvement in Domestic Tasks. HLW = Household Labour during the Workday. ^a^Point-biserial correlation. ^b^Tetrachoric correlation. *p < 0.05. **p < 0.01.

### Latent Variables Fit, Measurement Invariance and Factor Scores

The three factor CFA yielded an acceptable fit, with χ^2^ (41) = 285.49, *p* < .001; CFI = .95; TLI = .93; RMSEA = .073; SRMR = .06. The item “I have a lot of responsibility in my job.” from the job demands factor cross-loaded on the autonomy factor. As autonomy can also be a perk of having responsibilities at work (Glavin & Schieman, 2012), we added the cross-loading, which substantially improved the fit of the model, with χ^2^ (40) = 181.31, *p* < .001; CFI = .97; TLI = .96; RMSEA = .056; SRMR = .04.

The testing of the four levels of invariance showed that the latent variables met the requirements for strict invariance (ΔCFI < 0.01, ΔRMSEA < 0.015) between participants who answered the survey in French and in Dutch (see Table 3).

**Table 3:** Invariance Tests.


MODEL	χ^2^	DF	*P*	CFI	ΔCFI	RMSEA	ΔRMSEA

Configural	249.414	80	<0.001	0.966		0.058	

Metric	262.278	89	<0.001	0.965	0.000	0.055	0.003

Scalar	288.718	97	<0.001	0.962	0.003	0.055	–0.000

Strict	302.577	108	<0.001	0.959	0.003	0.055	0.000


The factor scores were reliable, relative to their latent variable counterpart, with reliabilities of .85, .77 and .87 for job demands, autonomy and work engagement while teleworking, respectively.

### Hypotheses testing

The multivariable fractional polynomials procedure demonstrated that there was a non-linear relationship between IDT and HLW. A transformation of IDT to a FP2 of powers (2, 3) fitted significantly better to the data than the linear term and FP1 (see Table 4). Thus, in our model, IDT should be implemented as x^2^ + x^3^ instead of x. The squared and cubic transformations of IDT are centered around their respective means (Royston & Sauerbrei, 2008). The other covariates did not display a non-linear relationship with HLW.

**Table 4:** Results of the Multivariable Fractional Polynomials Procedure.


INVOLVEMENT IN DOMESTIC TASKS	df	DEVIANCE	Δ DEVIANCE^a^	*p*	POWERS

FP2	4	1138.9	—	—	2, 3

FP1	2	1147.5	8.6	<.05	–0.5

Linear	1	1156.9	18.0	<.001	1


*Note*. FP2 = two-term fractional polynomial. FP1 = one-term fractional polynomial. ^a^Follows a χ_(df)_^2^ distribution.

The interaction tests did not show significant results for the interactions of gender with job demands, autonomy and IDT on HLW (see Table 5). The interaction coefficient between gender and job demands was not significant in Model 2 (*B* = 0.017, *SE* = 0.178, *W* = 0.09). In Model 3, the interaction coefficient between gender and autonomy (*B* = 0.258, *SE* = 0.159, *W* = 1.62), and the likelihood ratio test with Model 1 (LRT = 2.7 (1)) were almost significant, with *p* < 0.10. Similarly, in Model 4, the interaction coefficient between gender and work engagement while working remotely (*B* = 0.276, *SE =* 0.159, *W* = 1.73), and the likelihood ratio test with Model 1 (LRT = 3.0 (1)) were almost significant, with *p* < 0.10. In Model 5, the FP2 transformation of IDT was significant for women but not for men. Nonetheless, the interaction did not provide a significant improvement of model fit compared to Model 1 (LRT = 2.7 (2)). Additionally, Model 1 provided the lowest BIC value of the five models, confirming that adding an interaction term would overfit the data. Therefore, the moderation hypothesis H6 (a, b, c and d) could not be confirmed, and we considered Model 1 as our final model.

**Table 5:** Interaction Tests Between Gender and Job Demands (Model 2), Autonomy (Model 3), Work Engagement (Model 4), and IDT (Model 5) On HLW.


	MODEL 1				MODEL 2				MODEL 3				MODEL 4				MODEL 5			
				
	*B*	*SE*	*WALD*	*P*	*B*	*SE*	*WALD*	*P*	*B*	*SE*	*WALD*	*P*	*B*	*SE*	*WALD*	*P*	*B*	*SE*	*WALD*	*P*

Intercept	–3.81	0.494	–7.72	<0.001	–3.81	0.496	–7.69	<0.001	–3.83	0.497	–7.71	<0.01	–3.79	0.498	–7.61	<0.001	–3.68	0.508	–7.25	<0.001

**Focal variables**																				

Job demands	0.332	0.081	4.09	<0.001	0.321	0.156	2.038	<0.05	0.333	0.081	4.09	<0.001	0.333	0.082	4.08	<0.001	0.333	0.081	4.11	<0.001

Autonomy	–0.371	0.078	–4.78	<0.001	–0.371	0.078	–4.77	<0.001	–0.555	0.139	–3.99	<0.001	–0.371	0.078	–4.75	<0.001	–0.367	0.078	–4.71	<0.001

Work engagement	0.192	0.089	2.17	<0.05	–0.192	0.089	2.16	<0.05	0.200	0.089	2.24	<0.05	0.019	0.134	0.14	>0.05	0.193	0.089	2.18	<0.05

Gender	0.175	0.173	1.01	>0.05	0.172	0.177	0.97	>0.05	0.192	0.174	1.10	>0.05	0.160	0.175	0.91	>0.05	–0.106	0.247	–0.43	>0.05

IDT^2^	–0.112	0.022	–5.17	<0.001	–0.112	0.022	–5.16	<0.001	–0.114	0.022	–5.26	<0.001	–0.113	0.022	–5.21	<0.001	—	—	—	—

IDT^3^	0.009	0.002	4.62	<0.001	0.009	0.002	4.61	<0.001	0.009	0.002	4.70	<0.001	0.009	0.002	4.66	<0.001	—	—	—	—

**Gender × Job demands** (M2)	—	—	—	—	0.017	0.178	0.09	>0.05	—	—	—	—	—	—	—	—	—	—	—	—

**Gender × Autonomy** (M3)	—	—	—	—	—	—	—	—	0.258	0.159	1.62	>0.05	0.276	0.159	1.73	>0.05^†^	—	—	—	—

**Gender × Work engagement** (M4)	—	—	—	—	—	—	—	—	—	—	—	—	—	—	—	—	—	—	—	—

**Gender × IDT** (M5)																				

IDT^2^ (if Gender = Men)	—	—	—	—	—	—	—	—	—	—	—	—	—	—	—	—	–0.067	0.038	–1.76	>0.05^†^

IDT^3^ (if Gender = Men)	—	—	—	—	—	—	—	—	—	—	—	—	—	—	—	—	0.005	0.004	1.44	>0.05

IDT^2^ (if Gender = Women)	—	—	—	—	—	—	—	—	—	—	—	—					–0.138	0.027	–5.10	<0.001

IDT^3^ (if Gender = Women)	—	—	—	—	—	—	—	—	—	—	—	—					0.011	0.002	4.68	<0.001

**Control Variables**																				

Remote work frequency	–0.141	0.075	–1.87	>0.05^†^	–0.141	0.076	–1.87	>0.05^†^	–0.143	0.076	–1.88	>0.05^†^	–0.147	0.076	–1.94	>0.05^†^	–0.132	0.075	–1.76	>0.05^†^

Age	0.053	0.008	6.87	<0.001	0.053	0.008	6.86	<0.001	0.054	0.008	6.86	<0.001	0.053	0.008	6.82	<0.001	0.054	0.008	6.87	<0.001

Cohabitants	–0.068	0.068	–1.00	>0.05	–0.068	0.068	–1.00	>0.05	–0.069	0.068	–1.01	>0.05	–0.071	0.069	–1.04	>0.05	–0.064	0.069	–0.93	>0.05

*Log Likelihood*	–563.8				–563.8				–562.4				–562.2				–562.4			

*LRT ^a^*					0 (1)				2.7^†^ (1)				3.0† (1)				2.7 (2)			

*BIC*	1199.3				1206.5				1203.8				1203.4				1210.9			

*McFadden’s R^2^*	0.113				0.113				0.115				0.115				0.115			


*Note*. IDT = Involvement in Domestic Tasks. LRT = likelihood ratio test. IDT^2^, IDT^3^ are the two-terms transformation (square and cubic) of the Involvement in Domestic Tasks variable, resulting from the multivariable fractional polynomials procedure. Gender is coded as 0 = Men, 1 = Women. ^a^follows a χ^2^ distribution. ^†^*p* < 0.10.

Model 1 provided adequate fit to our data. The Hosmer-Lemeshow test was not significant, with *Ĉ* (14) = 12.12, *p* = 0.596 and the Stukel test yielded a non-significant LRT of χ^2^ (2) = 0.213, *p* = 0.899. The accuracy of the model was acceptable, with AROC = 0.732.

Results showed that autonomy decreased by 8.8% the probability of HLW, with AME [95%CI] = –0.088 [–0.118, –0.054]. We hypothesized that autonomy would be positively related to HLW, thus H1 was not confirmed. In the contrary, job demands significantly increased the probability of HLW by 5.4%, with AME [95%CI] = 0.054 [0.026, 0.082]. As we expected that job demands would be negatively related to HLW, H2 could not be confirmed. Similarly, work engagement while remote working increased the probability to do HLW by 2.7% (AME [95%CI] = 0.027 [0.006, 0.045]). We expected that work engagement while teleworking would be negatively related to HLW, thus H3 was not confirmed.

**Figure 2:**
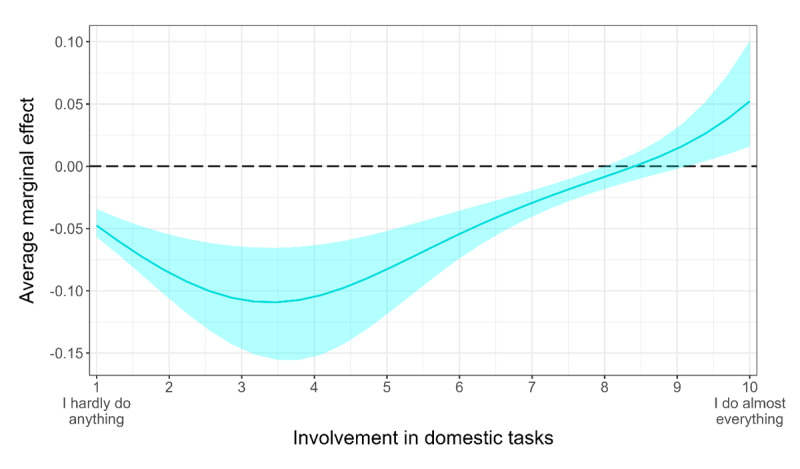
Average Marginal Effects and 95%CIs of IDT on HLW, Conditional on the Values of IDT.

Regarding IDT, H4 posited that IDT would be positively related to HLW. IDT was significantly and non-linearly associated with HLW. Low to above average levels of IDT (1-*I hardly do anything* to 7) decreased between 10.7% (level 3) and 2.9% (level 7) the probability of HLW. However, respondents who reported doing almost everything in their household (level 10) were 5.2% more likely to do HLW, with AME [95%CI] = 0.052 [0.016, 0.106]. Thus, H4 is partially supported, as only the highest level of IDT was positively related to HLW, whereas lower levels of IDT were negatively related to HLW. See Figure 2 for the plot of AMEs and their 95%CIs given the level of IDT.

Finally, gender was not significantly related to HLW, with AME [95%CI] = 0.026 [–0.016, 0.072]. Therefore, H5 was not confirmed, as we expected that women would be more likely than men to do HLW when working remotely.

For reference, the control variable age had a significant relationship with HLW (AME [95%CI] = 0.007 [0.005, 0.009]). Cohabitants had no significant relationship with HLW (AME [95%CI] = –0.009 [–0.025, 0.009]). Remote work frequency had no significant relationship with HLW, with AME [95%CI] = –0.018 [–0.039, 0.001].

## Discussion

Using border theory (Clark, 2000), this article investigated how autonomy, job demands, work engagement while teleworking and IDT could affect the borders of the work domain when teleworking, by doing HLW. Moreover, from the gender roles theory (Eagly et al., 2000), we studied whether HLW would be more likely among women than men, and if autonomy, job demands and IDT had a different relationship with the performance of HLW, depending on gender.

The findings suggest that greater autonomy is linked to a lower probability of doing HLW. On the contrary, greater job demands and work engagement when working remotely are related to a higher probability of doing HLW. These three results are in contradiction with hypotheses 1 to 3, which stated that autonomy would be positively related to HLW, and that both job demands and work engagement would be negatively related to HLW.

Moreover, by using multivariable fractional polynomials, a method commonly employed in medical research but rarely in work psychology (Nikolaeva et al., 2015), we found that IDT was non-linearly associated with HLW. Specifically, the respondents who did almost all domestic tasks by themselves had a higher probability of doing HLW, whereas others had lower probability of doing HLW. This partly confirms H4, which stated that IDT would be positively related to HLW.

Finally, we found no significant effects of gender. Women were not more likely than men to do HLW (H5), and men were not less likely than women to do HLW when having high job demands (H6a), high autonomy (H6b) or high IDT (H6c).

The negative relationship between autonomy and HLW somewhat challenges the managerial perception that autonomy when working remotely could lead workers to be distracted, notably by household demands, and to interrupt their work (Aloisi & De Stefano, 2022; Clouet, 2022). This result could imply that remote workers with more autonomy compress or expand their workhours, depending on the respective needs of their work and family domain; nonetheless, they would not let household labour flow to the work domain. They could be adapting to the demands of both domains, without having to deal with the self-regulatory cost of task interruptions and task switching (Leroy et al., 2020). Moreover, previous studies have shown that workers who benefit from autonomy, notably when working remotely, can feel a heightened responsibility to be productive, which can translate into taking less breaks, working for longer hours and not allowing household labour to interrupt their work (Clar-Novak, 2025; Tammelin et al., 2017; Vayre et al., 2022).

The positive relationship of job demands with HLW could be due to an increased likelihood of working outside of traditional workhours, especially when working remotely, where borders are more permeable (González-Fernández et al., 2025; Wöhrmann & Ebner, 2021). Nonetheless, family demands must still be tended to at some point of the day. Thus, it could imply that high job demands can lead to doing HLW, due to longer workdays ending up being interrupted by family demands. For workers that are exposed to high job demands, working from the office could leave them with limited time and energy to tend to family demands once they get back home, potentially leading to work-to-family conflicts (Michel et al., 2011). In this context, working remotely could help them to reduce some aspects of work-to-family conflicts, by being able to tend more easily to family demands despite the high level of job demands. However, it could also put them at risk of family demands interrupting their work, leading to family-to-work conflict (Michel et al., 2011).

A similar pattern occurred for work engagement while working remotely. Our results suggest that workers who are highly engaged in their work when working remotely tend to do HLW. Although it contradicts previous findings (e.g. Chen & Huang, 2016; Halbesleben et al., 2009), a key difference to keep in mind between these studies and our results is the work setting of the respondents: working from the office *versus* working from home. A plausible interpretation of these diverging results can be drawn from the multiple roles theory (Marks, 1977), which posits that having multiple roles is not necessarily strenuous, granted that these roles are not mutually exclusive and can be integrated. Whether the roles are exclusive or not does not depend on their respective activities, but rather on the social institutions and cultural norms delineating said roles (Marks, 1977). While western societies tend to segregate work and family roles, and put the emphasis on the work role (Eagly et al., 2000; Marks, 1977), remote work, by weakening the borders between the work and family domain (Gálvez et al., 2019; Wöhrmann & Ebner, 2021), has the potential of somewhat challenging these norms. Therefore, from a multiple role theory perspective, the energy that characterizes work engagement (Bakker & Demerouti, 2008) could also benefit HLW when working remotely.

Regarding the non-linear association between IDT and HLW, it seems to imply that HLW is done out of necessity, when workers are likely to become overloaded with domestic tasks. Indeed, workers become more likely to do HLW only at the highest level of IDT. This is coherent with previous studies showing that, similarly to how job demands can spillover to the family domain, having to take care of most of household labour can spillover to the work domain (Chatot et al., 2023; S. Wang & Cheng, 2024). The lower probability of doing HLW when workers at lower levels of IDT seem to indicate that when workers do not have to deal with almost all domestic tasks by themselves, they can maintain work domain borders that are impermeable to household labour.

Lastly, the social roles ascribing women to be “homemakers” and men to be “breadwinners” did not increase the likelihood of women to do HLW when working remotely, relatively to men (Eagly et al., 2000), nor did it affect the relationships of job demands, autonomy, work engagement while working remotely or IDT with HLW. These results somewhat contradict previous research, although the disruption brought by the Covid-19 pandemic on the practice of remote work should be taken into consideration. Indeed, studies investigating household labour in the context of remote work have been using quantitative and qualitative data either from prior the Covid-19 pandemic (e.g. Chung & Van Der Horst, 2020; Eddleston & Mulki, 2017; Gálvez et al., 2019; Genadek & Hill, 2017; S. Wang & Cheng, 2024) or from during the Covid-19 pandemic (Chatot et al., 2023; Collins et al., 2021; Hanzl & Rehm, 2023; Parry, 2025). Prior to the Covid-19 pandemic, remote work practice was relatively scarce (Duchêne et al., 2024) and could be considered to be family-friendly (Chung & Van Der Horst, 2020). As such, women who had access to remote work could have been more inclined to do HLW in this work setting or chose to work remotely because it allowed them to better manage a high amount of household labor. However, the Covid-19 pandemic lockdowns substantially increased household labor, especially for mothers, to the point where HLW became a necessity rather than a choice for women who worked remotely and who lived with their male partner or who were parents (Clouet, 2022; Kley & Reimer, 2023; Parry, 2025). Such an amount of household labour is not representative of what is usually experienced in a “normal” societal context, with functioning schools and childcare services. Nonetheless, now that remote work is increasingly becoming a norm in organizations (Eurofound and European Commission Joint Research Centre, 2024), the past experience of being overburdened by household labour during the Covid-19 pandemic may have incited women to maintain stronger borders for the work domain when working remotely. Moreover, perhaps a substantial part of the workers who have gained access to remote work since the Covid-19 pandemic aims to and has developed strategies to maintain strong borders for their work domain. An alternative explanation could be that the performance of household labour is becoming more balanced between men and women in heterosexual relationships. Nonetheless, the organization and planification of household labour continues to be mainly taken care of by women (Daminger, 2020).

## Limitations and Future Research

This study is not without limitations. The different scale formats of the study variables, from binary to 4–, 5–, and 7-levels Likert scales, help reducing some aspects of common method bias in a cross-sectional design (Podsakoff et al., 2024). Nonetheless, presence of common method bias cannot be ruled out. Moreover, such a design does not allow us to infer the directionality of the hypothesized relationships. For instance, it may well be that respondents feel overwhelmed and report a high level of job demands because they perform domestic tasks during the workday. Thus, future studies should introduce a time lag between work characteristics, personal attitudes, and HLW to establish the directionality of these relationships as well as to control common method bias (Podsakoff et al., 2024).

The assessment of IDT through a single item, although useful for an exploratory study, remains limited. This item requires respondents to give an estimation of their general involvement in domestic tasks but does not give information about the level of involvement in specific tasks, and the frequency of said tasks. Indeed, domestic tasks are also gendered, with male-typed tasks (e.g. repairs, gardening) typically being less frequent than female-typed tasks (e.g. cooking, laundry, cleaning), that often occur daily (Quadlin & Doan, 2018). Therefore, men and women could have been referring to different type of tasks when responding to this item, which could explain the lack of a significant difference between men and women regarding the relationship between IDT and HLW. Moreover, IDT does not reflect the cognitive work necessary to the planification and organization of household labour, which are mainly taken care of by women in heterosexual relationships (Daminger, 2020). Therefore, future studies should consider assessing IDT with a diversity of items pertaining to specific domestic tasks, as well as the planification and organization of household labour.

Although we found that autonomy decreased the probability to do HLW, this relationship could stem from socio-economic differences. Workers who have higher work autonomy tend to belong to higher occupational classes and have higher wages (Gil-Hernández et al., 2024) and are thus more likely to outsource household labour (Deuflhard, 2024). Workers who outsource household labour could be less likely to do HLW, as they have less household labour to tend to. This could explain why autonomy was negatively related to HLW in our study. Future studies should consider controlling for the outsourcing of household labour.

Furthermore, the research has taken place in Belgium, which may contribute to explain some of the divergences between the results from this study and the existing literature. Indeed, most research surrounding remote work and its relationship with the work and life domains has taken place in Anglo-Saxon countries (Blom et al., 2025), where the work devotion schema is well established, social (including family) policies are minimal and family services are privatised (Williams et al., 2013; Woods, 2018). These aspects contribute to the maintenance of strongly gendered social roles (“breadwinning” men and “homemaking” women). In the case of Belgium, stronger family policies, such as paid maternity and paternity leaves or higher public spending on childcare (Rizzi & Rees, 2023), may have helped alleviating some aspects of gender inequalities in the division of household labour. Therefore, future studies should consider making cross-country comparisons to assess whether the results from the present study are generalizable to different cultural contexts.

## Data Availability

The data supporting the findings of this study can be made available by the corresponding author upon reasonable request.
